# Exploiting GPUs in Virtual Machine for BioCloud

**DOI:** 10.1155/2013/939460

**Published:** 2013-04-24

**Authors:** Heeseung Jo, Jinkyu Jeong, Myoungho Lee, Dong Hoon Choi

**Affiliations:** ^1^Department of Information Technology, Chonbuk National University, 567 Baekje-daero, Deokjin-gu, Jeonju-si, Jeollabuk-do 561-756, Republic of Korea; ^2^Department of Computer Science, Korea Advanced Institute of Science and Technology, 291 Daehak-ro, Yuseong-gu, Daejeon 305-701, Republic of Korea; ^3^Department of Computer Science and Engineering, Myongji University, 116 Myoungji-ro, Cheoin-gu, Yongin, Gyeonggi-do 449-728, Republic of Korea; ^4^Korea Institute of Science and Technology Information (KISTI), 245 Daehak-ro, Yuseong-gu, Daejeon 305-806, Republic of Korea

## Abstract

Recently, biological applications start to be reimplemented into the applications which exploit many cores of GPUs for better computation performance. Therefore, by providing virtualized GPUs to VMs in cloud computing environment, many biological applications will willingly move into cloud environment to enhance their computation performance and utilize infinite cloud computing resource while reducing expenses for computations. In this paper, we propose a BioCloud system architecture that enables VMs to use GPUs in cloud environment. Because much of the previous research has focused on the sharing mechanism of GPUs among VMs, they cannot achieve enough performance for biological applications of which computation throughput is more crucial rather than sharing. The proposed system exploits the pass-through mode of PCI express (PCI-E) channel. By making each VM be able to access underlying GPUs directly, applications can show almost the same performance as when those are in native environment. In addition, our scheme multiplexes GPUs by using hot plug-in/out device features of PCI-E channel. By adding or removing GPUs in each VM in on-demand manner, VMs in the same physical host can time-share their GPUs. We implemented the proposed system using the Xen VMM and NVIDIA GPUs and showed that our prototype is highly effective for biological GPU applications in cloud environment.

## 1. Introduction

Virtualization technology has been widely adopted into computing systems to increase hardware resource utilization and reduce total cost of ownership (TCO). Virtualization technology enables multiple computing environments to be consolidated in a single physical machine. This consolidation brings efficient use of hardware resources and flexible resource provisioning to each computing environment [1]. In cloud computing, virtualization is key enabling technology because flexible resource provisioning is essential for unpredictable user demands.

Although virtualization adds an additional software layer over bare metal hardware, the overhead incurred by the additional layer has been reduced by various efforts. Hardware vendors have extended their architectures to support virtualization. In system software area, paravirtualization techniques, such as the Xen [2], reduce the performance gap between native environment and virtualized environment by slightly modifying operating systems in virtual machines (VMs). Due to the narrowed performance gap, the virtualization technology expands its coverage from cloud computing to high performance computing [3–5]. Recently, biological applications, which require high performance computing environment, are moving into cloud computing environment due to the narrowed performance gap and the advantage of flexible resource provisioning [6–8]. It is better to use elastic resources in cloud than to build one's own computing cluster in terms of TCO. 

Meanwhile, graphic processing units (GPUs) are recently exploited in high performance computing because a GPU provides one or two orders of magnitude faster computation than a CPU does. The computing capability of GPU has been rapidly improved for a decade. To cover vastly increased demands of 2D and 3D data processing, GPU embeds hundreds of computing cores in a single chipset. In addition, memory bandwidth in GPU is also widened to follow the increased computing power. For example, NVIDIA Tesla C2090, a state-of-the-art GPU device, is equipped with 512 computing cores, 6 GB of dedicated memory and 177 GB/s internal memory bus [9]. It provides 1331 GFLOPS for single precision floating point operation and 665 GFLOPS for double precision floating point operation. 

With the advance of GPU hardware, general purpose GPU computation on graphic processing units (GPGPU) has been emerging to exploit high performance computing capability of GPU not only for 3D graphic manipulation but also for general-purpose computation. The GPU programming framework such as the CUDA [10] and the OpenCL [11] provide application programming interfaces (APIs) of underlying GPU hardware, GPU programming model, and memory model. Due to the open APIs, many general-purpose applications can exploit fast GPUs for their computation-intensive applications [12]. 

Data copies between host memory and internal memory of GPU could be overhead of GPU computing. In GPU programming models, it is essential to store data on GPU-accessible memory for GPU to manipulate them. Accordingly, the data copies become a critical performance bottleneck of GPU computing. The data copy overhead, however, has been alleviated by making host memory GPU-accessible. In the application processing unit (APU) of AMD, CPU and GPU are integrated in a single chipset, and the two computing units share the same memory controller so that GPU can access the host memory. In addition, various studies have been conducted to lessen the data copy overhead. Despite of the data copy overhead, the performance improvement by GPU computing is significant in many research areas [10, 13–15].

With the trend of GPU computing in high performance computing and biological applications, virtualization needs to be incorporated in the GPU based high performance computing platforms. By providing virtualized GPUs to VMs in cloud computing environment, many biological applications will willingly move into cloud environment to reduce their expenses for computations. However, few studies have been done to use GPU computing in virtual machine environment, and most of them have limitations in terms of performance penalties by GPU virtualization overhead. Therefore, it is also important to minimize the overhead caused by GPU virtualization. 

In this paper, we propose a BioCloud system architecture that enables VMs to use GPUs in cloud environment. From the high performance computing power of GPUs, biological applications hosted in cloud can also show high performance while minimizing TCO of their computing infrastructure. The proposed system exploits the pass-through mode of PCI express (PCI-E) channel. By making each VM to be able to access underlying GPUs directly, applications can show almost the same performance as when those are in native environment. In addition, our scheme multiplexes GPUs by using hot plug-in/out device features of PCI-E channel. By adding or removing GPUs in each VM in on-demand manner, VMs in the same physical host can time-share its GPUs. 

The rest of the paper is organized as follows.  Section 2 introduces brief background and describes related work.  Section 3 presents the design and operation of our GPU virtualization and sharing mechanisms.  Section 4 demonstrates evaluation results and usability, and Section 5 discusses the superiority of our proposed system for biological applications. Finally, Section 6 concludes our work.

## 2. Background and Related Work

### 2.1. VMM and GPU Virtualization

Virtualization provides an illusion, a VM, to its hosted operating system. By multiplexing underlying hardware resources, a physical host can consolidate multiple VMs simultaneously. The core of virtualization technology is virtual machine monitor (VMM) that is in charge of multiplexing hardware resources such as CPU, memory, and I/O devices to multiple VMs. The common role of VMMs is to provide virtualized resources albeit their implementations diverse from emulation of virtual devices to hardware-assisted virtualization. 

Hardware-level assists to virtualization take overhead out of a VMM. Previously, a VMM either emulates the behavior of virtualized devices or incorporates with operation systems in VMs. The overhead of these approaches affects the system performance as compared to native environment. To reduce this overhead, hardware vendors extended their CPUs to support virtualization. The Intel VT [16] and the AMD-V [17] unburden the VMM overhead of CPU and memory virtualization. In addition, the Intel VT-d [18] and the AMD-Vi [19] support device virtualization so that operating systems in VMs can directly access I/O devices without security concerns. Despite of these technology advances, GPU has limitations for virtualization by itself. Since a GPU is in charge of manipulating graphic data, a large amount of data should be transferred from a VM to a GPU device. Since the amount of data is too large, emulation, one of methods to virtualize a device, is inefficient for GPUs. 

In GPU virtualization, there have been some related previous research such as the gVirtuS [20], the GViM [21], and the vCUDA [22]. These ultimately aim at providing the flexible sharing of a GPU among VMs taking performance degradation lying down. Therefore, they have the similar architecture that host operating system or VMM manages the overall operations and privileges of GPU. In the case of the gVirtuS based on KVM, one of VMMs working on top of host operating system provides a mechanism to access GPU based on the communication between virtual device drivers on host operating system and VM for each. The GViM and the vCUDA are based on the Xen, a VMM on bare-metal, and similarly use virtual device drivers between VMs and VM0 which is in charge of I/O. Although these GPU virtualization architectures, which are based on virtual device drivers, can enhance the sharing of GPUs among VMs, there are two critical limitations as below.

- Reimplementation and low flexibility of GPU APIs: for sharing of GPU among VMs, the management of GPU is concentrated on host operating system or VMM, and the communication between virtual device drivers is highly dependent on the implementation of them. Actually, they have to reimplement all GPU APIs, and this limits flexibility and portability. For example, if a version of GPU APIs is updated or modified, the virtual device drivers should be reengineered according to the changes. Even more, VMs in a physical host should use the same GPU APIs and version as the implemented virtual device drivers.
- High performance overhead: the architectures on related work adopt the fine-grained time-sharing technique among VMs to share GPU, but these largely depredate the overall performance of GPU by increasing the communication traffic between virtual device drivers and system bus. Moreover, the response times of GPU are not uniform due to the scheduling of VMs. According to the papers, they show 10–40% performance degradation.

In order to overcome these limitations, our scheme uses the direct pass-through approach to use GPU in virtualized environment [23]. By using the direct pass-through approach, operating systems in VMs can exclusively and directly access underlying GPU devices. Accordingly, the performance penalty when VMM involves in arbitrating GPUs can be eliminated.

### 2.2. GPU and Biological Application

The term GPU was mentioned by NVIDIA Corporation first, when it announced a new graphic controller named GeForce in 1999. In the early 1990s, graphic controllers in general PCs were in charge of simply translating computation results of CPU to visual characters on monitors. After the mid of 1990s, the role of graphic controller started to change into manipulating rich multimedia contents. Multimedia contents usually require lightning effects and texture mapping in order to make the contents more realistic. These computations burden CPU, thus an additional coprocessor, like GPU, is needed to lessen the computation load for multimedia processing in CPU. 

**Figure 1:**
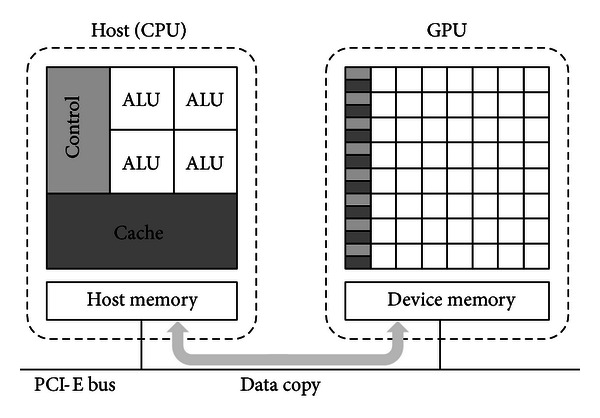
The architecture of a GPU equipped machine on PCI-E channel.

Although the architecture of GPU is similar to that of CPU, GPU has enhanced processing parallelism as shown in Figure 1. In the figure, CPU has a few high performance arithmetic and logic units (ALUs) and the large region of chipset is assigned to internal caches. The reason for this composition is to improve performance in task-level parallelism. On the other hands, GPU consists of many small ALUs optimized for graphic data processing in a single chipset. Since the characteristic of computations on GPU is highly data parallel, multiple data can be calculated by the same arithmetic operations. Accordingly, GPU outperforms the CPU in terms of parallel processing on the same computation with multiple data [24]. 

Since computations supported by GPU are specialized to graphical data manipulation, general-purpose computations cannot be easily ported into GPU. To address this obstacle, various general-purpose GPU frameworks have been built. The CUDA of NVIDIA and the OpenCL of Khronos Group are most representative frameworks in the area of general-purpose high performance computing. These frameworks provide an extended C language so that non-graphic-friendly programmers can intuitively translate their computation logics into GPU-friendly ones. 

From the consensus between GPU performance improvement and easy programming APIs, biological applications prevalently start to use GPUs for their computations. Especially, computations requiring a large amount of data, such as next generation sequencing and protein simulation, are proper targets to exploit GPUs. Manavski and Valle suggested a GPU implementation of Smith-Waterman sequence alignment [6], and Vouzis and Sahinidis transformed the BLAST tool to a GPU based application, named the GPU-BLAST [7]. The barraCUDA [25] and the G-aligner [26] are also kinds of short sequence alignment tool with GPU acceleration. 

## 3. GPU Virtualization

### 3.1. Overview

Our approach for virtualizing and sharing of GPUs is based on the GPU direct access scheme in our previous work [23]. The previous work exploits the PCI-E direct pass-through mechanism of GPUs in order to reduce the virtualization overhead and to increase the GPU API flexibility. Since the direct pass-through approach can minimize the interference incurred by VMM, VMs can achieve bare-metal performance. Moreover, since each VM can use their own GPU APIs, it can be freed from the reengineering and modification of GPU APIs. 

The direct pass-through approach in virtualized system should be supported by input/output memory management unit (IOMMU) hardware feature. Similar to a traditional memory management unit (MMU) which translates CPU-visible virtual addresses to physical addresses, IOMMU takes care of mapping device-visible I/O addresses to physical addresses and also provides memory protection from misbehaving devices.

**Figure 2:**
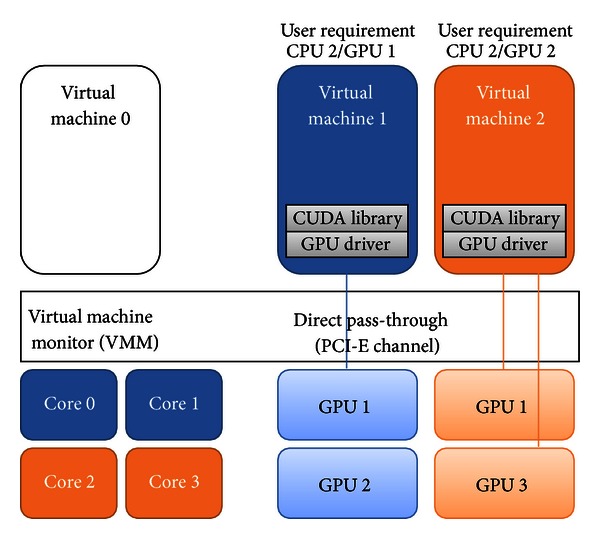
The system architecture of direct pass-through GPU virtualization.

Figure 2 shows the system architecture diagram of the proposed scheme, the GPU virtualization using direct pass-through. The system we suppose has a privileged control VM, generally called VM0, to interface with VMM and control other user VMs. Note that VM0 denotes the privileged control VM, and guest VMs denote other VMs of users. In the system, each GPU is attached to PCI-E channels and VMM passes the control of PCI-E channel and GPU to VMs. Each VM has its own GPU API and GPU device driver, and it can access and control GPU without the intervention of VMM. 

However, the direct pass-through mechanism has a limitation that the sharing of GPU among VMs is not possible. For example, once a GPU is allocated to a VM at booting time of the VM, it cannot be deallocated until the VM halts. To address this problem, we extend our previous work to have the coarse-grained sharing mechanism based on the hot plug functionality of PCI-E channel. The sharing mechanism enables VM0 to allocate GPUs to VMs and deallocate GPUs from VMs while the VMs are running. To enhance the utilization of GPUs, we add two features: (1) allocating GPUs when a VM actually requires GPUs to compute its data and (2) deallocating GPUs immediately after the computation.

The hot plug-in/out mechanism that is adopted in this research is a mechanism to install or remove PCI devices on online. To utilize the hot plug mechanism in virtualized systems, VMM needs the functionality of IOMMU. The system we suppose has GPU installed on a PCI-E channel, and a GPU can be allocated or deallocated by using the PCI-E channel hot plug-in/out. Due to this mechanism, the users of VMs can utilize virtualized GPUs in the same way with native GPUs. 

**Figure 3:**
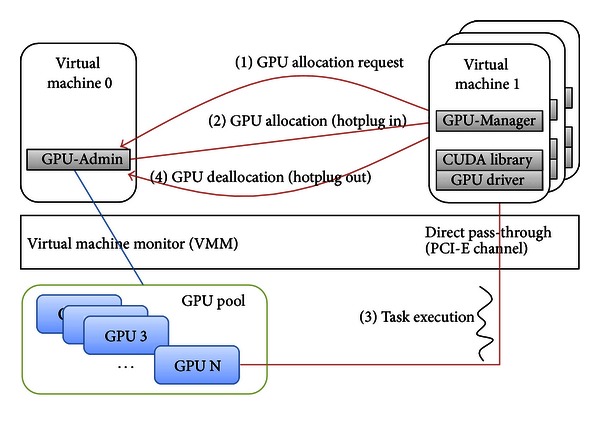
The overall coarse-grained GPU sharing mechanism sequences.

Figure 3 shows the overall coarse-grained GPU sharing mechanism. First, (1) each guest VM requests GPU allocation to the VM0 when it needs GPU computation. Then, (2) the VM0 checks the GPU pool which has all GPUs installed on the host machine to find an available GPU. If there is an available GPU in the GPU pool, the VM0 plugs in the GPU into the requested VM. (3) The guest VM processes its job, and (4) the GPU is revoked from the guest VM after the end of computation using the hot plug-out message. 

**Figure 4:**
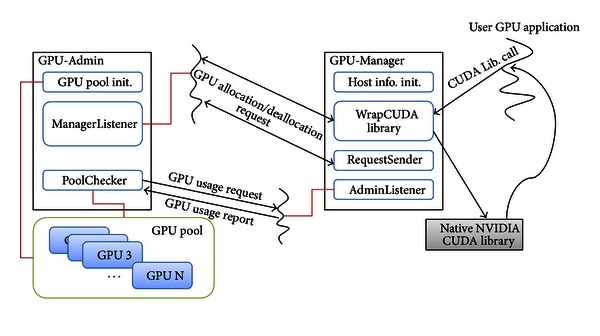
The detailed modules and operations of the GPU-Admin and the GPU-Manger.

In this mechanism, largely, we have two main considerations. One is when to allocate and deallocate GPUs. Although the users of guest VMs can directly request the allocation and deallocation of GPUs, it might decrease usability. It is inconvenient to allocate GPUs manually before invoking a GPU application. We need to provide more convenient interfaces. The other is how to prevent excessive occupation of GPUs by VMs. It is crucial to increase the overall utilization of GPUs in a system. Therefore, we need a compulsory revoking mechanism when a GPU of a guest VM is not utilized for computation. Then, the reclaimed GPU can be used for other guest VMs. For this mechanism, we designed and implemented the GPU-Admin module in the VM0 and the *GPU-Manager* module in the guest VMs, respectively, as shown in Figure 4. The detailed operations of them are described in the following subsections.

### 3.2. GPU-Admin

The GPU-Admin is a daemon process in the VM0 and performs allocation and deallocation of GPUs according to the request of the GPU-Managers. It also takes in charge of compulsory revocation of unused GPUs. For this responsibility, it periodically checks the status of GPUs allocated for guest VMs and deallocates them if GPUs are not actually used for computation. 

In the initial stage of the GPU-Admin, it identifies the number of GPUs installed on a virtualized system and records the PCI-E channel information and the slot ID of each GPU. Using this information, the GPU-Admin registers all GPUs into the GPU pool for later management. All of the registered GPUs are initialized and stay in available state, and one of them is allocated via the GPU-Admin when a guest VM requests a GPU. At this moment, the GPU-Admin stores the virtual machine identification (VMID) and IP address of the guest VM to be referred for usage checking and compulsory revoking operations. After the initialization of GPU pool, the GPU-Admin creates two worker threads: the ManagerListener which handles the requests of the GPU-Manager and the PoolChecker to prevent unnecessary GPU occupation of VMs. 

#### 3.2.1. ManagerListener

The ManagerListener worker is a part of GPU-Admin to accept and handle the requests from guest VMs. The message types of GPU-Managers in guest VMs are five, and the corresponding reactions are as follows.

1. GPU allocation request by a user: it is the case that the user of a guest VM explicitly requests the allocation of GPU for computation. Responding to this request, the ManagerListener picks an available GPU from the GPU pool and allocates it into the requested guest VM via hot plug-in mechanism.
2. GPU deallocation request by a user: on contrary to (1), this message is to explicitly deallocate an allocated GPU of guest VM by a user. The ManagerListener deallocates the GPU from the guest VM and registers it again into the GPU pool after reinitialization of the GPU.
3. GPU allocation request by the WrapCUDA library: when a user of a VM invokes a GPU application without an allocated GPU, the WrapCUDA library detects the situation and automatically requests GPU allocation to the GPU-Admin. This request message is delivered implicitly and transparently without recognition of users. The handling of this message is the same with the case (1).
4. GPU deallocation request by the WrapCUDA library: before finishing GPU application, if it calls several specific CUDA library call, the WrapCUDA library requests the deallocation of GPUs implicitly. This mechanism is also performed without the intervention of users. Responding to this message, the ManagerListener deallocates the GPU similarly to the case (2).
5. Disconnection from WrapCUDA library: from the case (3) to (4), the ManagerListener and the WrapCUDA library keeps their connection. If the connection is disconnected for any reasons such as halting or finishing the GPU application, the ManagerListener recognizes it and then deallocates the GPU of the guest VM.

In our prototype, we use TCP/IP network communication method between the GPU-Admin and the GPU-Manager to utilize the virtue of its well-defined and concrete interfaces. Since the communication messages are not incurred frequently, its overhead is negligible. The hot plug-in/out operations of GPUs by the ManagerListener are performed via the management interface of the Xen. 

In the case that there is no available GPU in the GPU pool, the allocation request is inserted into the waiting queue of the ManagerListener, and the response is blocked until a GPU become available by being deallocated from other guest VMs. If more than one allocation request messages are waiting in the waiting queue, the ManagerListener prioritizes them according to their waiting time.

#### 3.2.2. PoolChecker

The major role of the PoolChecker is to find the allocated GPUs to guest VMs and decide that they are currently used for computation or not. We add this mechanism to prevent the situation that a guest VM excessively occupies GPUs while it does not actually utilize GPUs for computation. Due to this mechanism, the system can achieve higher overall utilization of GPUs by inhabiting unnecessary occupation of GPUs. 

The PoolChecker works periodically. When triggered, it checks the GPU pool to search GPUs already allocated to guest VMs and sends a message requesting usage report of GPU to each GPU-Manager of guest VMs. On receipt of this message, each GPU-Manager investigates the usage of its own GPU and replies to the PoolChecker. The PoolChecker tracks the utilization of each GPU and deallocates it forcibly, if it has not been used for computation for a while. This GPU utilization check mechanism of the PoolChecker is a last resort to prevent useless occupation of GPUs by guest VMs. In most cases, where the users of guest VMs are not evil, the unused GPUs are immediately reclaimed by the deallocation message or the disconnection message of the WrapCUDA library. 

Our prototype sets the trigger period of the PoolChecker as five seconds and the reclaim time limit as ten seconds. These configured values are decided intuitively because we assume that the users of VMs might not need GPUs currently, if the GPUs are not used for computation more than ten seconds. The time-out value of the PoolChecker is easily reconfigurable by editing the configuration file of the PoolChecker. 

### 3.3. GPU-Manager

The GPU-Manager is a module working in a guest VM to provide interfaces allocating or deallocating GPUs and report the utilization of its GPU responding to the request of the PoolChecker in the GPU-Admin. The interfaces to request GPU allocation and deallocation can be divided in two. One is a transparent and implicit interface, which is requested by the WrapCUDA library when a user starts and finishes a GPU application. The other is an explicit interface, which is performed by a user action of guest VM.

In the initialization stage of the GPU-Manager, it identifies its hostname, local IP address, and GPU-Admin IP address to communicate with the GPU-Admin for allocation and deallocation of GPUs. The GPU-Manager consists of three parts: the AdminListener thread which handles the request of the PoolChecker, the WrapCUDA library hooking the API calls of the native CUDA library to support the implicit allocation or deallocation, and the RequestSender to provide the explicit user interface.

#### 3.3.1. AdminListener

AdminListener thread works similarly to the ManagerListener of GPU-Admin. It opens a specified port and waits the GPU usage report request. After allocation of GPUs, the PoolChecker sends periodical requests to check the utilization of GPUs, and the AdminListener responds to this request. To get the utilization of its GPUs, the AdminListener searches the usage of GPU driver module in the proc filesystem, a virtual filesystem presenting process and system information. At least, if more than one GPU application utilizes GPU hardware, the driver module works and the usage of the driver module increases. Therefore, sending this information, the AdminListener reports the usage of GPUs to the PoolChecker. Then, the GPU-Admin can deallocate the GPUs of guest VMs based on this criteria, if it decides that the GPUs are not used for computation.

#### 3.3.2. WrapCUDA Library

The WrapCUDA library of GPU-Manager is to allocate and deallocate GPUs automatically. To increase the usability of GPUs, the WrapCUDA library enables GPU allocation and deallocation without the intervention of users, when a GPU application starts and finishes. 

The WrapCUDA library is implemented in a shared library and is preloaded using LD_PRELOAD environment variable after the startup of guest VMs. It is in charge of dynamic allocation of GPU when a user starts a GPU application even if one's VM does not have a GPU. A GPU application should use a specified programming framework such as the CUDA to interface GPU, and several APIs of them are generally called to probe and initialize its GPU in the initial stage of the application. For example, the cudaGetDeviceCount() function to get the number of GPUs installed on system and the cudaMalloc() to allocate heap memory on GPU are representatives. The WrapCUDA library hooks these kinds of several native CUDA library calls. If a GPU application calls the wrapped CUDA library functions to access GPU, they are redirected to the WrapCUDA library which implements the same function due to LD_PRELOAD environment value. Then, the WrapCUDA library checks whether its guest VM has a GPU and requests GPU allocation if the guest VM has no GPU. After a GPU is allocated, the function of WrapCUDA library executes the native function of the CUDA library. 

Similarly, a GPU application calls several CUDA APIs to release the resource of GPU at finishing time, and the WrapCUDA library catches these calls before transferring to the native CUDA library for deallocation of GPU from its VM. Although a GPU application might not call these resource release APIs by implementation, the WrapCUDA library can deallocate the GPU of VM immediately, since the connection between the WrapCUDA library and the GPU-Admin is disconnected after the halt of a GPU application. Additionally, as mentioned in Section 3, we also implement the PoolChecker mechanism, which checks the status of GPU and deallocates it after several seconds to prevent the occupation of GPUs unnecessarily. 

In order to interpose the API calls of GPU applications, the WrapCUDA library has to define API functions to catch and embed the implementations of them. These might not be a burden to implement the WrapCUDA library, since the number of APIs to start GPU application is limited to several ones and the patterns to develop a GPU application are regularized. Actually, our prototype implements the API functions listed in Table 1, and the top three functions can cover all the 84 GPU application examples and several biological GPU applications used in the evaluation section. Even more, for the implementation of a function call in the WrapCUDA library, we used seventeen C language code lines as shown in Algorithm 1. This engineering overhead is negligible for an experienced programmer and can be applied to other GPU programming frameworks besides the CUDA programming framework. 

#### 3.3.3. RequestSender

To run GPU applications without the WrapCUDA library mechanism, the users of VMs can explicitly request the allocation and deallocation of GPUs. The RequestSender module provides the explicit interfaces. In this case, the GPU explicitly allocated via the RequestSender is specially handled as an exception for the PoolChecker. Therefore, it is not deallocated automatically until the user requests deallocation of the GPU via the RequestSender interface. Since this mechanism enables a user of VM to monopolize GPUs in a system, it should be allowed to trusted users when they need continuous and reliable GPU computing environment. 

## 4. Evaluation

### 4.1. Overview

We used the Xen VMM for implementing and evaluating the prototype of the proposed BioCloud architecture. For GPU computation, we used the NVIDIA GPUs and the CUDA programming frameworks. The host machine specification is summarized in Table 2. Briefly, the machine is equipped with Intel Xeon E5620 CPU, four NVIDIA Quadro FX 3800 GPUs, and 24 GB of main memory. Each GPU is installed on PCI-E (2nd generation) channel in the host machine. 

The main goals of the prototype evaluation are (1) the GPU virtualization overhead of our scheme, when GPU applications run in virtualized environment as compared to those in native environment, (2) the latencies of GPU hot plug-in/out in VMs, and (3) the benefits by our scheme. 

### 4.2. Virtualization Overhead

In this section, we compare the proposed virtualization scheme with others which are mentioned in the related work and measure the biological application execution time when each application runs in native environment and in virtualized environment using our scheme. 

**Figure 5:**
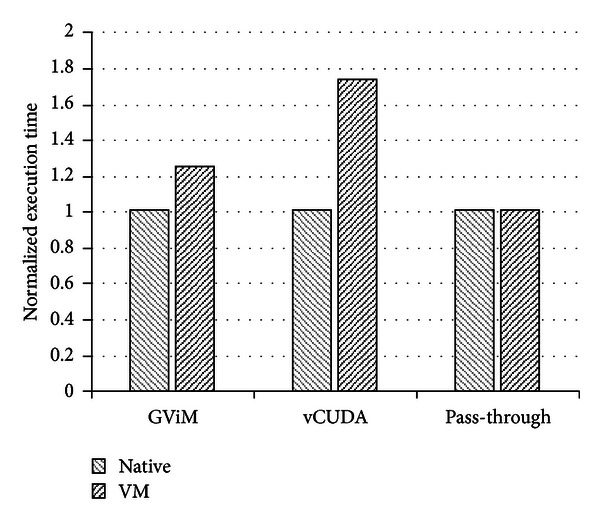
The performance comparison with other schemes.

Figure 5 shows the execution time comparison of BlackScholes application among the GViM, the vCUDA, and our scheme. Unfortunately, we cannot replay all the three schemes (gVirtuS, GViM, vCUDA) because their hardware and software configuration are too outdated. Instead, we borrowed the evaluation results compared native environment based on their papers. Note that the gVirtuS scheme is omitted because it does not evaluate BlackScholes application. Although their evaluation environments are all different from each other, we can focus on the gap between native and VM and identify that the execution time overhead of our scheme is less than 0.5%, while those of others are 25~73%. Because the other two schemes are based on the virtual device driver mechanism and focused on the sharing of a GPU among VMs, their overheads are not negligible. 

**Figure 6:**
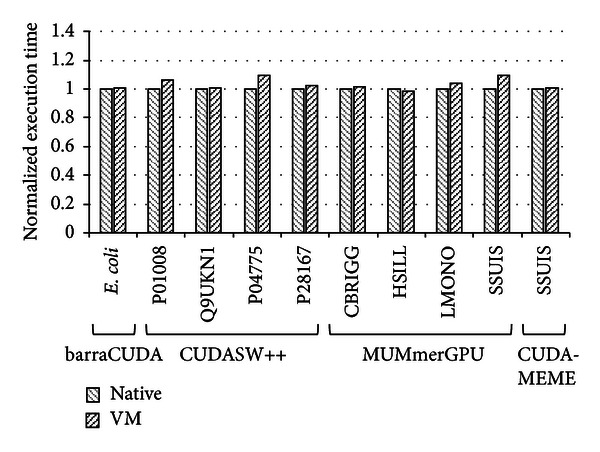
The performance evaluation using bioapplications.

Figure 6 is the evaluation results of several biological applications. In this evaluation, we ran the barraCUDA [25], CUDASW++ [27], MUMmerGPU [28], and CUDA-MEME [29]. The *x*-axis denotes the workloads and biological applications, and the *y*-axis is normalized execution time which includes disk I/O, CPU computation, and GPU computation. The overhead of our scheme is 3% on average, and the maximum overhead case is the MUMmerGPU with the SSUIS workload by 10%. From our analysis, the most part of overhead is caused by the disk I/O of virtualized system to read workload data files, not by the GPU virtualization. Except the disk I/O time, the GPU computation time is the same as that in the native environment. Comparing the result of Figure 5 which does not include the disk I/O overhead, we can confirm that the direct pass-through GPU virtualization mechanism shows the least overhead and highly useful for long running biological applications. 

### 4.3. Sharing Effect

#### 4.3.1. GPU Allocation and Deallocation Overhead

Our scheme uses GPU hot plug-in/out to provide sharing of GPUs among VMs. In this evaluation, we measure additional time overhead to hot plug-in/out of GPUs to a VM.  Table 3 shows the time for allocating and deallocating a GPU in a VM. Each value is the average of ten attempts. As shown in the table, the allocation time and the deallocation time are 1.3 seconds, identically. Considering general biological applications take from tens of minutes to hundreds of minutes, this allocation and deallocation time penalty is negligible compared to the total execution time of general biological applications.

#### 4.3.2. Effect of Real Workload

The main role of our scheme is to make a GPU directly accessible and to coordinate the ownership of a GPU among VMs for time sharing. In order to reveal the effect of GPU sharing in our scheme, we measured the application execution time when multiple VMs share a finite number of GPUs. In this evaluation, we used the barraCUDA application that performs next generation sequencing. Each of the barraCUDA applications performs five-million read mappings of *E. coli* genome data. One execution of the application takes 18 seconds in average. One workload in a VM runs the barraCUDA application five times with sleep time between consecutive runs. The sleep time models the behavior of a user that interprets the result of a previous run and adjusts parameters for the next run. We varied the sleep time to 9, 18, and 36 seconds, and each are 50%, 100%, and 200% of the application execution time, respectively. During this sleep time, an allocated GPU to a VM is returned to the GPU-Admin and could be reallocated to another VM for sharing. Accordingly, we can expect performance improvement by reducing the idle time of GPUs. 

**Figure 7:**
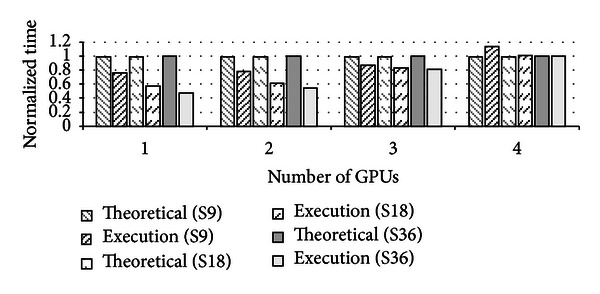
The GPU sharing effect using the barraCUDA.

Figure 7 shows the evaluation results with four VMs and varying the number of GPUs from one to four. The sleep time of each evaluation is denoted in the parentheses of each legend. For example, *Execution(S9)* denotes that the sleep time is 9 seconds. *Execution* denotes the real execution of four VMs while *Theoretical* denotes the theoretical execution time in four VMs when a GPU(s) is exclusively used by the VM. Hence, the time is the same as the execution time when four workloads run in a single VM sequentially with the given number of GPUs. Albeit the theoretical execution time is not realizable without our mechanism, the time is used for comparison purpose. The execution time in our scheme is normalized to the theoretical time with the same configuration of sleep time and the number of GPUs, for each. 

In case of *Execution(S9)*, the sleep time after each completion of application is 9 seconds. When four VMs share one GPU, our scheme shows reduced total execution time by 20% as compared to the theoretical time. When the sleep time increases, our scheme reduces the total execution time because the increased sleep time naturally increases the probability to share the GPU during the sleep time. In case of *Execution(S36)* with one GPU, our scheme shows reduced total execution time by 53%. As the number of GPUs increases, the performance benefit of our scheme reduces. When the number of VMs is the same as the number of GPUs, our scheme shows no performance benefit. But, we believe that the number of VMs requiring GPUs will be more than the number of GPUs installed in a physical host, since multiple applications and VMs will be consolidated in a single physical machine in the cloud computing environment. 

#### 4.3.3. Waiting Time

**Figure 8:**
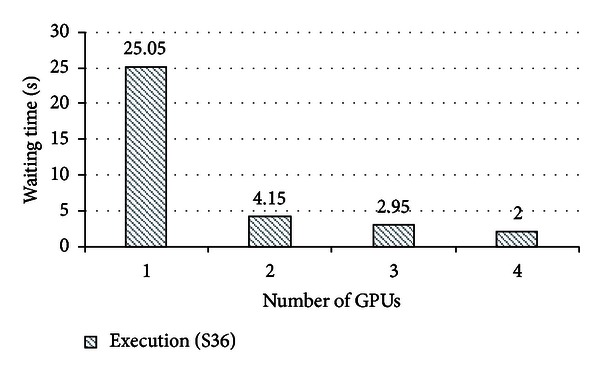
The average waiting time of VMs.

Finally, we measure the waiting time, the time to wait for allocation of a GPU, in the same evaluation. When there is no available GPU, a VM should be idle until a GPU becomes available. Accordingly, this additional waiting time can worse the overall execution time of each workload.  Figure 8 shows the average GPU waiting time with varying the number of GPUs from one to four. When the number of GPU is one, the average waiting time is 25.05 seconds. Although this waiting time could increase the execution time of a single workload, the overall performance is still improved as shown in Figure 7. In addition, when the number of GPU increases more than one, the average waiting time is significantly reduced to 4.15 seconds. Except the hot plug-in time, 1.3 seconds as shown in Table 3, a VM should wait 2.85 seconds on average when the number of GPU is two. This result indicates that if the number of GPUs is more than one, GPUs can be efficiently shared among VMs with negligible performance degradation.

The modeled scenario in the two previous subsections is synthetic and might be different case by case. Note that the benefit gap and the waiting time in these evaluations will diverse according to the running time and the sleep time of GPU applications. But, at least, we can confirm that our mechanism to virtualize and share GPUs works fine and is reasonably effective for biological GPU applications in BioCloud. 

## 5. Discussion

In this section, we discuss how our proposed architecture is suitable for biological applications in terms of memory and time of workloads in bioinformatics. 

One of major characteristics of workloads in bioinformatics is massively data-intensive computing. For example, in a protein sequence alignment workload, a large volume of gnome references are indexed by large-size hashing index (tens of GBs). The workload can perform better when the reference indices are (mostly) in GPU memory and most of them are filled in GPU's internal cache memory [26]. When multiple workloads share a single GPU device at fine-grained level (e.g., API-level multiplexing [20–22]), the GPU memory should be shared between multiple workloads. Accordingly, the memory size for each workload is inevitably reduced. Otherwise, when a GPU context switch occurs, the data in a GPU's memory should be replaced with the data for the next workload [30]. This data replacement leads to unnecessary and slow data copies between host memory and GPU memory (and memory copies between host memory and guest VM memory [22]). Since the bioinformatics workloads are highly data intensive, these penalties by memory sharing can result in performance degradation. 

Our scheme, however, does not cause those penalties. In our GPU virtualization architecture, a workload can fully exploit the memory in a GPU device while the workload is running. A GPU context switch only occurs after a currently scheduled workload finishes. Accordingly, our scheme can show better performance by eliminating those memory penalties.

The other characteristic to note is that bioinformatics workload usually takes a long time from several minutes to hundreds of minutes depending on the workloads [25, 28]. Therefore, when the API-level multiplexing schemes are used, fine-grained sharing of GPUs may result in thousands of context switches on a GPU in a second. This frequent context switch causes frequent flushes of warmed-up data in GPU internal caches and even in GPU memory [30, 31]. Although the batching GPU APIs [22] are aimed at reducing the frequent context switches, it cannot eliminate whole context switches in a long-time period (tens of seconds) so that flushing warmed-up data in GPU cache and GPU memory is inevitable. 

Our GPU-virtualization architecture can avoid the frequent context switches in a GPU device thereby showing better performance. Since a GPU is multiplexed at coarse-grained level (at workload), each scheduled workload can fully exploit the cache and memory in a GPU without unnecessary flushing of data until the workload finishes. When the context switch occurs, the previous workload never reuses the warmed-up data in those memory spaces because the workload is finished. As evidence, our architecture shows minimal performance degradation as compared to the other schemes.

## 6. Conclusions

For higher utilization of systems, the machine virtualization and cloud computing trend will prevail more and more. Besides, the high performance computing based on GPU also has proved its outstanding capabilities for general-purpose computation in many research areas. Especially, the biological applications are outstanding, since their computation can derive a large amount of benefit from many cores of GPUs. 

For biological GPU applications, we propose a cloud system to exploit GPUs in VM while multiplexing them among VMs and achieving almost the same performance as that with native use of GPUs. Considering the characteristics of bioinformatics workloads which have long execution time, our GPU virtualization mechanism is focused on high GPU computation throughput, rather than sharing GPUs among VMs. Although our prototype is based on the Xen VMM and NVIDIA GPUs, it can be easily ported into other implementations. In the evaluation section, we showed the effectiveness of our mechanism using a modeled scenario. Although the performance benefit and the waiting time can diverse in case by case, we believe that our scheme is highly effective for biological computation using GPUs in cloud environment.

## Figures and Tables

**Algorithm 1:**
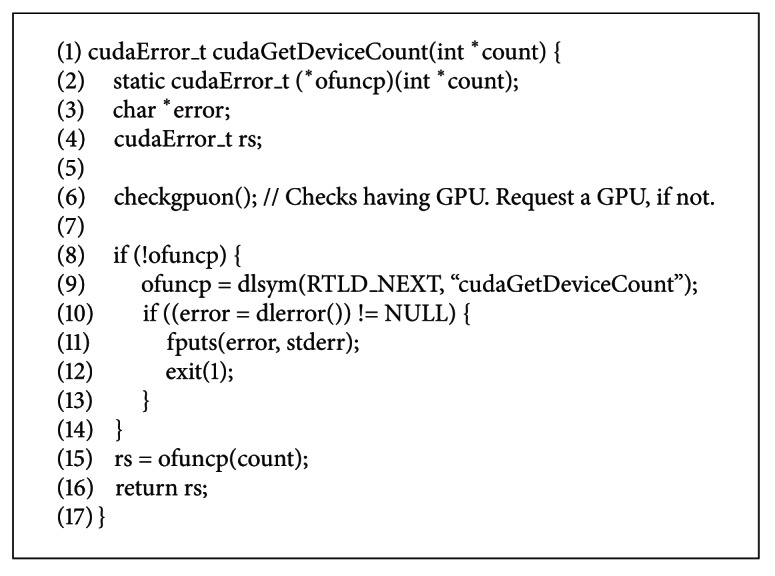
An example implementation of the WrapCUDA library function.

**Table 1:** The functions that are wrapped by the WrapCUDA library.

GPU allocation call	GPU deallocation call
cudaGetDeviceCount ( )	cudaThreadExit ( )
cudaGetDevice ( )	
cudaMalloc ( )	
cudaDeviceReset ( )	
cudaChooseDevice ( )	
cudaDeviceSynchronize ( )	

**Table 2:** The specifications of evaluation system.

Device	Specification
CPU	Intel(R) Xeon(R) E5620 (2.40 GHz)
Chipset	Intel(R) 5520
Memory	DDR3 1333 MHz (24 G)
PCI slot	PCI Express Gen2, 4 EA
GPU	NVIDIA Quadro FX 3800, 4 EA

**Table 3:** The time to hot plug-in/out of PCI-E channel.

Operation	Time (second)
GPU allocation (hot plug-in)	1.3 ± 0.1
GPU deallocation (hot plug-out)	1.3 ± 0.1

## References

[1] Meyer DT, Aggarwal G, Cully B Parallax: virtual disks for virtual machines.

[2] Barham P, Dragovic B, Fraser K Xen and the art of virtualization.

[3] Garg SK, Yeo CS, Anandasivam A, Buyya R (2011). Environment-conscious scheduling of HPC applications on distributed Cloud-oriented data centers. *Journal of Parallel and Distributed Computing*.

[4] Um J-H, Park SB, Seo JH, Choi DH Design of bio-cloud service for genomic analysis based on virtual infrastructure.

[5] Qiu X, Ekanayake J, Beason S Cloud technologies for bioinformatics applications.

[6] Manavski SA, Valle G (2008). CUDA compatible GPU cards as efficient hardware accelerators for Smith-Waterman sequence alignment. *BMC Bioinformatics*.

[7] Vouzis PD, Sahinidis NV (2011). GPU-BLAST: using graphics processors to accelerate protein sequence alignment. *Bioinformatics*.

[8] Meng Z, Li J, Zhou Y, Liu Q, Liu Y, Cao W (2011). CloudBLAST: an efficient mapreduce program for bioinforrnatics applications. *BMEI*.

[9] NVIDIA http://www.nvidia.com/docs/IO/105880/DS_Tesla-M2090_LR.pdf.

[10] Manavski SA CUDA compatible GPU as an efficient hardware accelerator for AES cryptography.

[11] Khronos OpenCL Working Group (2008). *The OpenCL 1. 0 Specification*.

[12] GPGPU http://gpgpu.org/.

[13] Cook DL, Keroymytis AD (2006). *Cryptographics: Exploiting Graphics Cards for Security*.

[14] Cook DL, Ioannidis J, Keromytis AD, Luck J CryptoGraphics: secret key cryptography using graphics cards.

[15] Govindaraju NK, Larsen S, Gray J, Manocha D A memory model for scientific algorithms on graphics processors.

[16] Uhlig R, Neiger G, Rodgers D (2005). Intel virtualization technology. *Computer*.

[17] AMD virtualization: introducing AMD virtualization. http://www.amd.com/virtualization/.

[18] Hiremane R (2007). Intel virtualization technology for directed I/O (Intel VT-d). *Technology@Intel Magazine*.

[19] AMD I/O virtualization technology (IOMMU) specification.

[20] Giunta G, Montella R, Agrillo G, Coviello G (2010). A GPGPU transparent virtualization component for high performance computing clouds. *Euro-Par 2010 Parallel Processing*.

[21] Gupta V, Gavrilovska A, Schwan K GViM: GPU-accelerated virtual machines.

[22] Shi L, Chen H, Sun J VCUDA: GPU accelerated high performance computing in virtual machines.

[23] Jo H, Lee M, Choi DH (2013). GPU virtualization using PCI direct pass-through. *Applied Mechanics and Materials*.

[24] Lee VW, Kim C, Chhugani J Debunking the 100X GPU vresus CPU Myth: an evaluation of throughput computing on CPU and GPU.

[25] Klus P, Lam S, Lyberg D (2012). BarraCUDA-a fast short read sequence aligner using graphics processing units. *BMC Research Notes*.

[26] Lu M, Tan Y, Bai G, Luo Q (2012). High-performance short sequence alignment with GPU acceleration. *Special Issue on Data Intensive Escience of Distributed and Parallel Databases*.

[27] Liu Y, Maskell DL, Schmidt B (2009). CUDASW++: optimizing Smith-Waterman sequence database searches for CUDA-enabled graphics processing units. *BMC Research Notes*.

[28] Schatz MC, Trapnell C, Delcher AL, Varshney A (2007). High-throughput sequence alignment using graphics processing units. *BMC Bioinformatics*.

[29] Liu Y, Schmidt B, Liu W, Maskell DL (2010). CUDA-MEME: accelerating motif discovery in biological sequences using CUDA-enabled graphics processing units. *Pattern Recognition Letters*.

[30] Kato S, McThrow M, Maltzhan C, Brandt S Gdev: first-class GPU resource management in the operating system.

[31] Kato S, Lakshmanan K, Rajkumar R, Ishikawa Y TimeGraph: GPU scheduling for real-time multi-tasking environments.

